# Inhibition of a Snake Venom Metalloproteinase by the Flavonoid Myricetin

**DOI:** 10.3390/molecules23102662

**Published:** 2018-10-16

**Authors:** Lina María Preciado, Jeffrey Comer, Vitelbina Núñez, Paola Rey-Súarez, Jaime Andrés Pereañez

**Affiliations:** 1 Programa de Ofidismo/Escorpionismo, Facultad de Ciencias Farmacéuticas y Alimentarias, Universidad de Antioquia UdeA, Calle 70 No. 52-21, Medellín 050010, Colombia; vitelbina.nunez@gmail.com (V.N.); ofidpa@gmail.com (P.R.-S.); 2 Institute of Computational Comparative Medicine, Kansas State University, Manhattan, KS 66506, USA; jeffcomer@gmail.com; 3 Escuela de Microbiología, Universidad de Antioquia, UdeA, Calle 70 No. 52-21, Medellín 050010, Colombia

**Keywords:** flavonoids, free-energy calculations, local tissue damage, molecular dynamics simulation, snake venom metalloproteinase

## Abstract

Most of the snakebite envenomations in Central and South America are caused by species belonging to *Bothrops* genus. Their venom is composed mainly by zinc-dependent metalloproteinases, responsible of the hemorrhage characteristic of these envenomations. The aim of this study was to determine the inhibitory ability of ten flavonoids on the in-vitro proteolytic activity of *Bothrops atrox* venom and on the hemorrhagic, edema-forming and myonecrotic activities of Batx-I, the most abundant metalloproteinase isolated from this venom. Myricetin was the most active compound, exhibiting an IC${}_{50}$ value of 150 μM and 1021 μM for the inhibition of proteolytic and hemorrhagic activity, respectively. Independent injection experiments, with a concentration of 1600 μM of myricetin administered locally, immediately after toxin injection, demonstrated a reduction of 28±6% in the hemorrhagic lesion. Additionally, myricetin at concentrations 800, 1200 and 1600 μM promoted a reduction in plasma creatine kinase activity induced by Batx-I of 21±2%, 60±5% and 63±2%, respectively. Molecular dynamics simulations coupled with the adaptive biasing method suggest that myricetin can bind to the metalloproteinase active site via formation of hydrogen bonds between the hydroxyl groups 3’, 4’ and 5’ of the benzyl moiety and amino acid Glu143 of the metalloproteinase. The hydroxyl substitution pattern of myricetin appears to be essential for its inhibitory activity. Based on this evidence, myricetin constitutes a candidate for the development of inhibitors to reduce local tissue damage in snakebite envenomations.

## 1. Introduction

Snakebites are an important public health problem affecting approximately 1.8–2.2 million people annually, resulting in 81,000–138,000 deaths [1]. In Latin America, the *Bothrops* genus (Viperidae) inflicts the vast majority of these bites, among which the lance-head vipers *B. asper* and *B. atrox* are medically relevant in Central and South America [2,3]. These envenomations are characterized by local pathological alterations associated with edema, myonecrosis, dermonecrosis, blistering, hemorrhage and systemic alterations such as coagulopathies, acute renal failure and cardiovascular shock which occur in moderate and severe cases [3]. This particular array of local and systemic alterations is mostly induced by the action of different enzymes, such as snake venom metalloproteinases (SVMPs), phospholipases A${}_{2}$ and serine proteases [4,5,6]. The SVMPs represent one of the most abundant components of the snake venoms of Viperidae family [7]. These enzymes are large multi-domain proteins which are classified into three major classes (P-I, P-II and P-III) based on the presence of various domains and their organization [8]. They are responsible for the hemorrhage, local edema, myonecrosis, inflammation and dermonecrosis induced by viperid snake envenomations [9,10].

The intravenous administration of animal-derived (mostly horse or sheep) antivenoms is the only effective treatment of snakebite envenomation. Clinical investigations have established that antivenoms are generally highly effective in the neutralization of toxins responsible for systemic effects such as coagulopathy and hemodynamic disturbances [11]. Nevertheless, antivenom efficacy is constrained by its limited capacity to neutralize local tissue damage induced by snake venoms. This problem is not due to a lack of neutralizing antibodies in antivenoms, but rather due to the extremely rapid development of local pathology that makes it difficult for neutralizing antibodies to access the area before irreversible damage occurs [12,13]. For this reason, it is important to develop alternative venom inhibitors, either synthetic or natural, in order to complement the action of antivenoms, particularly in the neutralization of local tissue damage.

Flavonoids are secondary plant metabolites classified into six groups: flavonol, flavanone, isoflavone, flavone, flavan-3-ol, and anthocyanin [14]. These compounds have been reported to possess antioxidant, hepatoprotective, anti-inflammatory, anticancer and antiviral activities [15,16]. In addition, the ability of different groups of flavonoids to inhibit the hemorrhagic activity of whole snake venoms or isolated SVMPs has been described. For instance, gallocatechin, isoquercitrin, pinostrobin, and quercetin-3-O-rhamnoside isolated from different plant species have been shown to inhibit hemorrhagic activity of different viperid and elapid venoms and some isolated SVMPs [17,18,19,20]. Similarly, the ability of flavonoids to inhibit human matrix metalloproteinases (MMPs) has been demonstrated [21,22]. The phenolic nucleus of flavonoids is able to form multiple interactions with proteins: hydrogen bonds, hydrophobic interactions, metal chelation and π–π stacking interactions [23].

**Figure 1:**
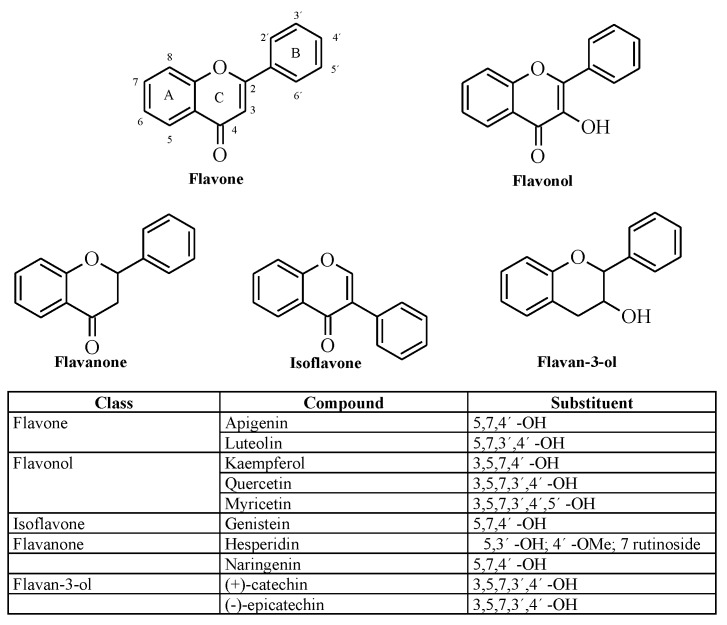
Chemical structure of evaluated flavonoids.

Throughout this study, different flavonoids were tested in order to determine their potential to inhibit the pharmacological activities of Batx-I, the most abundant PI-SVMP isolated from *B. atrox* venom. These compounds were chosen since they belong to different flavonoid classes (flavone, flavonol, flavonone, isoflavone and flavan-3-ol) and their hydroxylation patterns are distinct (Figure 1). In addition, molecular dynamics simulations with the enhanced sampling method adaptive biasing force (ABF) were carried out to calculate the potential of mean force and estimate the binding free-energy, as well as to reveal the atomic interactions between myricetin and the metalloproteinase.

## 2. Results

### 2.1. Inhibition of Proteolytic Activity

**Figure 2:**
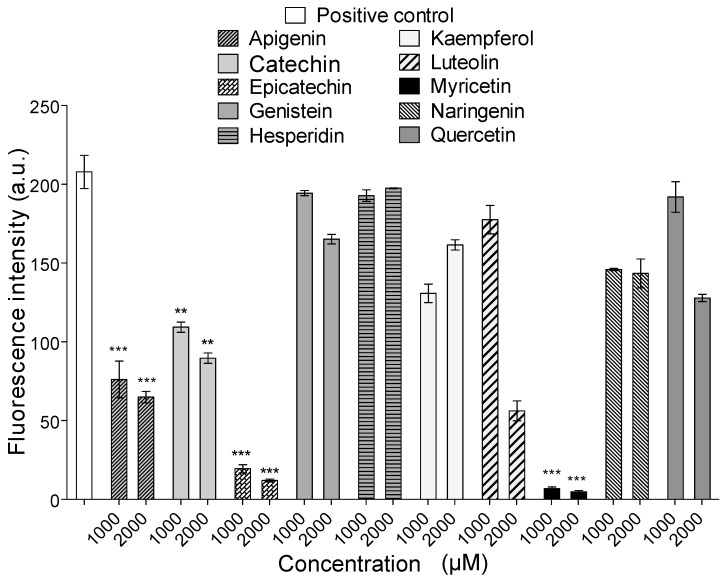
Inhibition of proteolytic activity of *Bothrops atrox* venom. *** represents statistical significant differences respect to positive control with p<0.001 and ** p<0.01

All flavonoids inhibited the proteolytic activity of *B. atrox* venom on fluorescein conjugates of gelatin (Figure 2). The most active compounds were the flavone apigenin and the flavanols epicatechin and myricetin. However, when these compounds were tested for their ability to inhibit the proteolytic activity of Batx-I, only myricetin showed inhibition higher than 50% at all tested concentrations (500, 1000 and 2000 μM). For this reason, myricetin was selected for the subsequent in vitro and in vivo experiments. The IC${}_{50}$ value of this flavanol for the inhibition of *B. atrox* venom proteolytic activity was 49.3 μM with a 95 % confidence interval of 47.5–51.2 μM.

**Figure 3:**
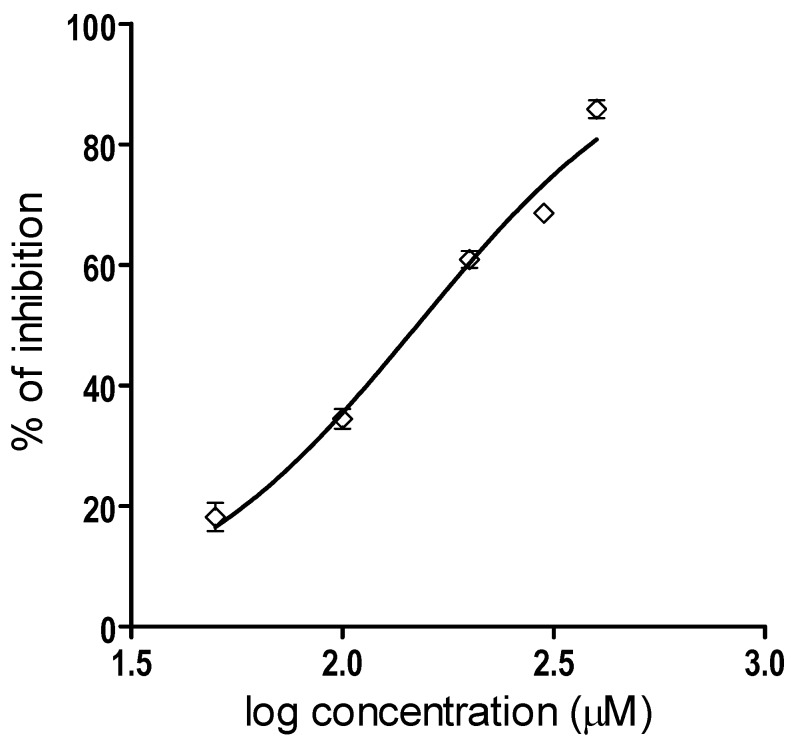
Graphical representation of the IC${}_{50}$ determination of Batx-I proteolytic activity inhibition. Five different concentrations of myricetin (ranging from 50 to 400 μM) were tested. Data are represented as % of inhibition and are expressed as mean values ± SEM (*n*= 3).

On the other hand, the IC${}_{50}$ value of myricetin for the inhibition of Batx-I proteolytic activity was 150.4 μM with a 95 % confidence interval of 138.2–163.7 μM (Figure 3). The determination of the IC${}_{50}$ values was done from a logistic-dose response curve.

### 2.2. Inhibition of Hemorrhagic Activity

**Figure 4:**
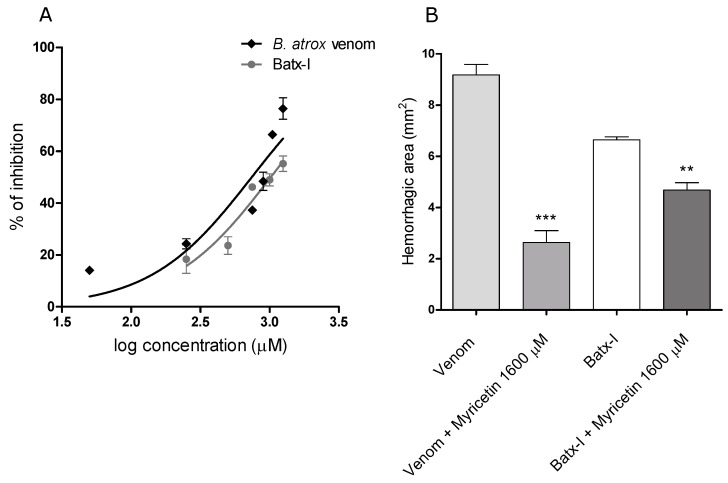
Inhibition of *B. atrox* venom and Batx-I hemorrhagic activity by myricetin. (**A**) graphical representation of the ${\mathrm{IC}}_{50}$ value determination on *B. atrox* venom and Batx-I hemorrhagic activity. Different concentrations of myricetin were tested in pre-incubation assays as was described in the materials and methods section. Data are represented as % of inhibition and are expressed as mean values ± SEM (*n*= 3); (**B**) inhibition of *B. atrox* venom and Batx-I hemorrhagic activity by myricetin with independent injection. The minimum hemorrhagic dose for *B. atrox* venom (6 μg /mouse) or Batx-I (30 μg /mouse) was injected intradermally into mice and subsequently a solution 1600 μM of myricetin was injected. Results are shown as mean ± SEM, *n*= 3. *** Represent statistical differences with p< 0.001 respect to *B. atrox* venom injection. ** Represent statistical differences with p< 0.01 respect to Batx-I injection.

Myricetin inhibited the hemorrhagic activity of the whole *B. atrox* venom and the isolated metalloproteinase Batx-I in pre-incubation assays in a concentration-dependent manner. The IC${}_{50}$ values were 743.9μM and 1021μM with confidence intervals (95%) of 600.3–922.0 μM and 868.4–1200.0 μM, respectively (Figure 4A). Independent injection studies showed that myricetin partially abrogated hemorrhagic activity when it was administered immediately after *B. atrox* venom or toxin injection (Figure 4B).

### 2.3. Inhibition of Edema-Forming and Myotoxic Activities

At all tested concentrations, myricetin did not induce a reduction of edema-forming activity of Batx-I. Similarly, injection of myricetin at a concentration of 1600 μM (negative control) did not induce edema-forming activity.

**Figure 5:**
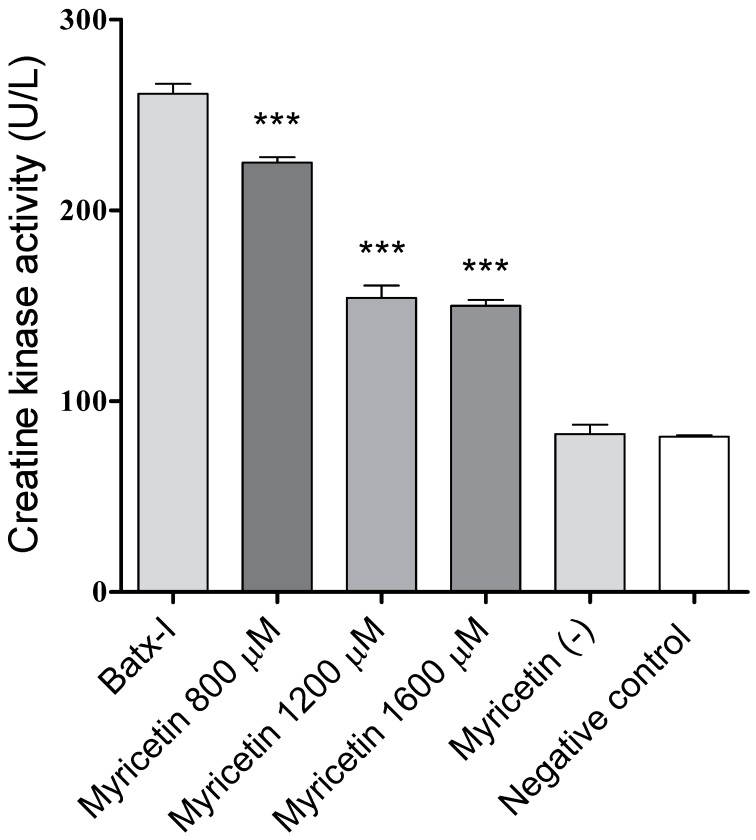
Inhibition by myricetin of myotoxic activity induced by Batx-I. *** Represents statistical differences with respect to Batx-I with *p*-value < 0.001. (*n*= 3).

On the other hand, myricetin induced significant inhibition of the myotoxic activity promoted by Batx-I (Figure 5), as made evident by a statistically significant reduction of plasma creatine kinase activity levels with respect to the positive control (enzyme alone) at all tested concentrations. The inhibitory activity at concentrations 1200 and 1600 μM were comparable (p>0.05). The plasma creatine kinase activities of mice injected with myricetin at 1600 μM or 1% dimethyl sulfoxide in sterile saline solution (negative control) were similar, showing that myricetin did not induce myotoxicity.

### 2.4. Intrinsic Fluorescence

**Figure 6:**
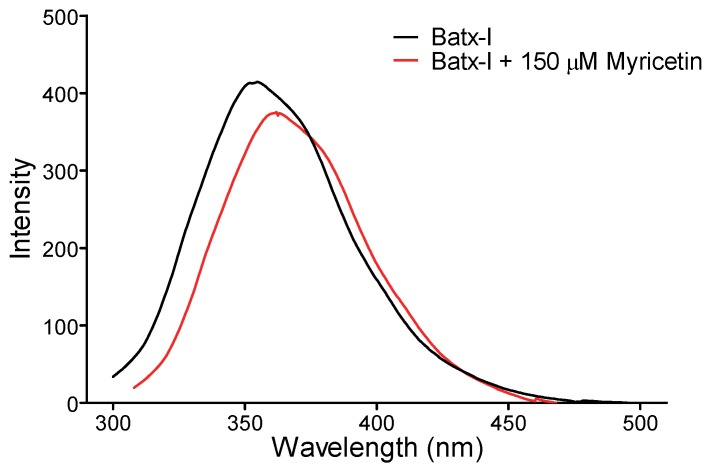
Intrinsic fluorescence spectrum of Batx-I in the presence or absence of 150 μM of myricetin. Results are shown as a mean of three independent experiments.

To determine the structural changes induced by myricetin on Batx-I, the intrinsic fluorescence of the enzyme in the presence and absence of myricetin at a concentration of 150 μM was recorded. Flavonol caused a moderate decrease in fluorescence intensity and displacement of the spectrum (Figure 6). These results suggest slight structural modifications to the enzyme and direct interaction of myricetin with the SVMP.

### 2.5. Computational Studies

**Figure 7:**
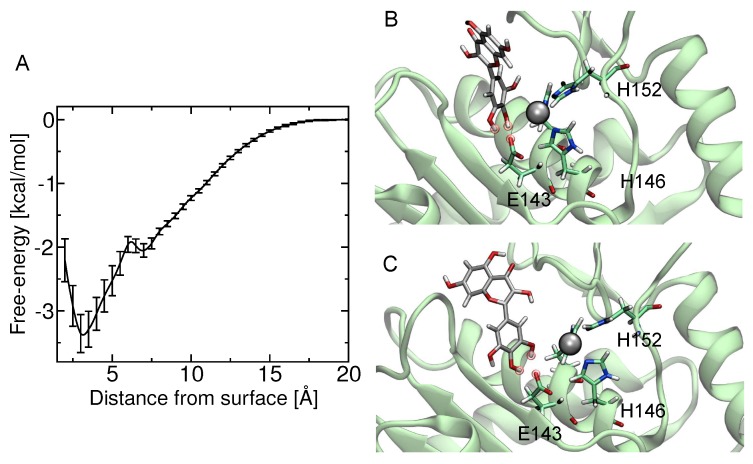
Free-energy calculation of myricetin binding to the active site of BaP1. (**A**) potential of mean force as a function of distance between the myricetin and metalloproteinase obtained from adaptive biasing force calculations; (**B**) image showing the proposed putative binding site for myricetin, with hydrogen bonds between hydroxyl groups at positions 3${}^{\prime }$ and 4${}^{\prime }$ of B-ring and one of the oxygens of the carboxylate group of the amino acid Glu143; (**C**) formation of hydrogen bonds between hydroxyl groups at positions 4${}^{\prime }$ and 5${}^{\prime }$ of B-ring and the amino acid Glu143.

The theoretical estimate of the standard binding free energy of myricetin and BaP1 was −1.73 kcal/mol with statistical uncertainty of 0.03 kcal/mol. The value of the standard dissociation constant was 60±3 mM. The free energy as a function of distance between the myricetin and metalloproteinase obtained from ABF calculations is shown in Figure 7A. This minimum free energy value is related to the formation of hydrogen bonds between hydroxyl groups on the B-ring (see Figure 1) (positions 3’, 4’ and 5’) and one oxygen atom from the carboxylate group of the amino acid Glu143 (Figure 7B,C). In addition, the presence of this hydrogen bond is in agreement with the pose obtained in docking calculations, which also predicted the formation of a hydrogen bond between the hydroxyl at position 4${}^{\prime }$ of B-ring and Glu143.

## 3. Discussion

Flavonoids consist of a large group of polyphenolic compounds having a benzo-γ-pyrone structure and are ubiquitously present in plants [16]. These compounds display antioxidant, anti-inflammatory, anti-mutagenic and anti-carcinogenic activities [24]. They are also potent inhibitors of several enzymes—for instance, xanthine oxidase [25], cyclo-oxygenase [26], protein kinases [27] and matrix metalloproteinases (MMPs) [21,28,29].

MMPs comprise a family of zinc proteolytic enzymes that are well known for their ability to degrade the extracellular matrix and take part in both normal and pathological processes [29]. These enzymes belong to the metzincin superfamily of metalloproteinases, which also contains SVMPs. Both groups have similar structural features in the metalloproteinase domain, including the zinc-binding motif. MMPs and SVMPs are also topologically equivalent and can be superimposed with significant variability found only in the loop regions connecting helices and strands [30]; consequently, inhibitors of MMPs, such as flavonoids, could have the potential to inhibit SVMPs.

Inhibition of the enzymatic activity of zinc metallopeptidases by flavonoids was initially reported by Soto et al [31]. Later, diverse studies demonstrated the ability of these compounds to inhibit MMP-1 [32], MMP-2 and MMP-9 [33], among others. Similary, gallocatechin and apigenin derivatives have been shown to antagonize the hemorrhagic activity of venoms and purified toxins from different Viperidae venoms [17,34]. Nonetheless, thus far, the most active recognized SVMP inhibitor is the peptidomimetic Batimastat, a compound that inhibits BaP1 proteolytic activity on biotinylated casein, with an IC${}_{50}$ value of 80 nM. It totally abrogates the hemorrhagic and dermonecrotic effects of BaP1 when it is administered immediately after metalloproteinase administration. Nevertheless, this compound has poor oral bioavailability and low solubility in physiological solutions [35], conditions that make its therapeutic application difficult and reinforce the importance of searching for alternative inhibitors that complement the action of antivenoms.

In this study, different flavonoids were tested for their potential to inhibit the proteolytic activity of whole *B. atrox* venom and its most abundant P-I type metalloproteinase, Batx-I. These compounds were selected since they belong to different flavonoid classes and exhibit diverse patterns for their hydroxyl groups. These substitution patterns are important for protein binding as they determine the ability to form hydrogen bonds. For the inhibition of proteolytic activity of whole *B. atrox* venom, apigenin, catechin, epicatechin, kaempferol, luteolin, myricetin and naringenin showed significant inhibition (p<0.05) with respect to the positive control at concentration 1 mM (Figure 2). Nevertheless, for the assays of inhibition of Batx-I proteolytic activity, only myricetin showed significant inhibition having an IC${}_{50}$ value of 150.4 μM. In addition, this compound showed an IC${}_{50}$ value of 49.33 μM for the inhibition of *B. atrox* venom proteolytic activity, demonstrating its ability to inhibit different metalloproteinases from *B. atrox* venom. It has been reported that the chemical structure of flavonoids, especially the hydroxyl substitution pattern, plays an important role in their biological activities [36]. Previous studies demonstrated that the existence of three hydroxyl groups at the A or B rings of the flavonoid structure determine their inhibitory activity on MMP-2 and MMP-9, using gelatin as a substrate [28]. Our results are consistent with this observation, since myricetin, in comparison with the other tested flavonoids, has the highest substitution of hydroxyl groups at the A and B-rings and exhibited the highest inhibitory activity on Batx-I gelatinase activity.

Proteolytic activity of SVMPs is a key step to induce extravasation of red blood cells from capillaries [37]. Thus, inhibition of SVMPs enzymatic activity can help to reduce their hemorrhagic activity. This observation is in agreement with our results, since myricetin also inhibited the hemorrhagic activity of Batx-I with IC${}_{50}$ of 1021 μM. However, some conditions of the in vivo assay could increase the IC${}_{50}$ with respect to the in vitro value, such as changes in the substrate, the presence of protein–protein interactions, tissue diffusion rate and/or competition between substrates [38]. On the other hand, independent injection of Batx-I and myricetin showed a reduction of 28±6% on dermal hemorrhagic lesion when this flavonoid was administered locally immediately after toxin injection. This low inhibition percentage could be explained by the fast development of local bleeding after metalloproteinase injection [39]. Similar results were obtained with an apigenin (flavone) analogue inhibiting local hemorrhage induced by *Echis carinatus* venom [34], and glycolic acid to inhibit the hemorrhagic activity of BaP1 [40].

Another common effect of envenomation by Viperidae snakes of the *Bothrops* genus is muscle tissue damage (myonecrosis) [41]. This effect is mainly induced by a group of highly basic proteins with phospholipase $A_{2}$ structure and, to a lesser extent, by SVMPs [42]. In this study, myricetin at concentrations of 800, 1200 and 1600 μM induced a reduction of 22.3%, 63.5% and 65.5%, respectively, of the plasma creatine kinase activity in mice injected with Batx-I. Similar studies have reported the ability of triterpenic acids to inhibit the myonecrosis induced by metalloproteinases, for instance, betulinic, oleanolic and ursolic acids decreased in a 27% the serum creatine kinase levels of mice injected with Batx-I [43]. Nonetheless, local edema was not significantly reduced (p>0.05) at all tested concentrations of myricetin. It has been reported that edema induced by SVMPs is a consequence of the extravasation produced by direct damage on microvessels, as well as degranulation of mast cells and the action of inflammatory molecules, such as, interleukin-1 and interleukin-6, which are released or synthesized in the course of envenomation [44]. These inflammatory events could be weakly inhibited by myricetin, which may result in the lack of neutralization of Batx-I edema forming activity. However, this hypothesis must be confirmed in future studies.

To investigate the interactions between the metalloproteinase and myricetin, molecular docking and molecular dynamics simulations implementing the ABF algorithm were carried out. Although the experiments were focused on Batx-I, a P-I type SVMP isolated from *B. atrox* venom from Colombia, its complete sequence remains unknown and no structural information is available. Thus, for the computational studies, a related P-I type metalloproteinase from *B. asper* venom from Costa Rica with a published X-ray structure, BaP1 [45], was used. This enzyme shows 100% homology with some internal peptides reported for a Batx-I sequence and the biological activities of both enzymes are comparable [46]. The result of the free-energy calculations is the potential of mean force as a function of the distance between the protein and ligand, ΔG(Z). The theoretical estimate of the standard binding free energy of myricetin and BaP1 was −1.73 kcal/mol, and the value of the standard dissociation constant was 59.8 mM. These values suggest that myricetin is a low-affinity ligand for the metalloproteinase, and is correlated with the results obtained for in vivo experiments, for which myricetin exhibited an IC${}_{50}$ value in the micromolar range for the inhibition of hemorrhage. The atomic interactions stabilizing the bound configuration (i.e., consistent with the minimum value of ΔG(Z)) revealed the formation of hydrogen bonds between the three hydroxyl groups located on the B-ring and the amino acid Glu143 of BaP1. Binding at this location may negatively affect the activity of the metalloproteinase, since the accepted mechanism for catalysis involves a glutamate residue (Glu143), which polarizes a zinc-bound water molecule, increasing its nucleophilic character. This polarized water subsequently cleaves the substrate peptide bond in an addition-elimination mechanism [47,48].

Computational studies are in agreement with the results of intrinsic fluorescence, which demonstrated that myricetin induces moderate changes in the enzyme fluorescence spectrum, indicating direct interaction. Similar effects were reported for the interaction and further inhibition of glycolic acid on BaP1 [40]. Thus, myricetin could be used as a lead compound for the further development of SVMP inhibitors.

## 4. Materials and Methods

### 4.1. Venoms and Toxins

*Bothrops atrox* venom was obtained by manual extraction from five adult specimens from the Department of Meta, in southeastern Colombia. These specimens are kept in captivity at the Serpentarium of Universidad de Antioquia. The venom was centrifuged at 800× *g* for 15 min, and supernatants were lyophilized and stored at −20 ${}^{{}^{\circ}}$C until used.

The inhibition assays were performed with the P-I type metalloproteinase Batx-I, purified from *B. atrox* venom by ion-exchange chromatography (CM-Sephadex) following the protocol described by [46]. The toxin purity was evaluated by reverse-phase high-performance liquid chromatography (RP-HPLC) and sodium dodecyl sulfate polyacrylamide gel electrophoresis (SDS-PAGE) [49] (Figure S1). Batx-I was dialyzed, lyophilized and stored at −20 ${}^{{}^{\circ}}$C.

### 4.2. Chemicals and Reagents

Apigenin, catechin, epicatechin, genistein, hesperidin, kaempferol, luteolin, myricetin, naringenin and quercetin were purchased from Sigma-Aldrich, Inc. (St. Louis, MO, USA). These compounds were diluted in 1% dimethyl sulfoxide in sterile saline solution (SS-D).

### 4.3. Animals

The experiments were done in Swiss-Webster mice (18–20 g body weight, 8 weeks old) following the guidelines of the Universidad de Antioquia Ethics Committee (License No. 90, August 2014).

### 4.4. Inhibition of Proteolytic Activity of B. atrox Venom or Purified Toxin

Fluorescein conjugates of gelatin were used for the detection of Batx-I inhibitors with the method described by Preciado et al. [43], using the EnzCheck Gelatinase/Collagenase assay kit (Molecular Probes Inc., Eugene, OR, USA). Briefly, 100μg of Batx-I or 200μg of *B. atrox* venom were mixed with 20 μL of DQ^TM^-gelatin (100 μg/mL) and different flavonoids concentrations for 60 min at $37{\;}^{{}^{\circ}}$C. The fluorescence intensity was measured for excitation at 485 nm and emission at 515 nm, at 1 min intervals for 60 min with a Synergy HT Multi-Mode Microplate Reader (BioTek Instruments, Inc.; Winooski, VT, USA). All samples were assayed in triplicate wells. The inhibition percentage of Batx-I reaction was calculated as follows:

(1) $$\mathrm{Inhibition}=\left(\frac{S_{control}-S_{sample}}{S_{control}}\right)\times 100,$$

where $S_{control}$ and $S_{sample}$ are the slopes of the graph fluorescence versus time of the control and sample, respectively.

### 4.5. Inhibition of Hemorrhagic Activity

Hemorrhagic activity was quantitatively determined following the method of Kondo et al. [50] with some modifications. Concentrations ranging from 100 to 1250 μM of myricetin and 30 μg of Batx-I or 6 μg of *B. atrox* venom were pre-incubated at 37 ${}^{{}^{\circ}}$C for 30 min. Later, groups of three mice were intradermally injected in the ventral abdominal region with 100μL of these mixtures. Batx-I alone (30μg) or *B. atrox* venom (6μg) were used as positive control for each experiment. After two hours, the specimens were euthanized by CO${}_{2}$ inhalation. The skin was removed, spread and fixed on a glass plate avoiding distortion of the original size. The cross-diameters of each hemorrhagic spot were measured from the visceral side of the skin through the glass plate. Saline solution and dimethyl sulfoxide 1 % (SS-D) and myricetin + SS-D (1250 μM) were used as negative controls. Additionally, independent injection experiments were performed in order to reproduce the circumstances of snakebites, when venom is injected and the inhibitor is administered after envenomation at the site of venom injection. Thus, groups of three mice were injected with 30μg i.d. of Batx-I or 6 μg of *B. atrox* venom. Then, myricetin (1600 μM) was injected immediately after toxin or venom injection. A higher dose than the IC${}_{50}$ value obtained in pre-incubation studies was chosen since this experiment will require a higher concentration of inhibitor, due to the rapid initiation of hemorrhage.

### 4.6. Inhibition of Edema-Forming Activity

Edema was evaluated following the methodology proposed by Lomonte et al. [51]. In addition, 30μg of Batx-I were injected into the right footpad in groups of three mice. Inhibition studies were performed with pre-incubation of Batx-I with myricetin at concentrations of 300, 450 or 600μM. Control groups were injected with 50μL of SS-D or myricetin. The progression of edema was evaluated with a caliper two hours after the injection.

### 4.7. Inhibition of Myotoxic Activity

The myotoxicity was determined according to the method described by Gutiérrez et al. [52]. Groups of three mice received an intramuscular injection (gastrocnemius) of 60 μg of Batx-I in 100 μL of SS-D. After three hours, a blood sample was collected from the caudal vein, in order to evaluate the creatine kinase (EC2.7.3.2) activity using a kinetic assay (Weiner Lab, CK-NAC UV-AA). Activity was expressed in U/L. For inhibition studies, mice were injected with 100 μL of toxin pre-incubated for 30 min at 37 ${}^{{}^{\circ}}$C with different concentrations of myricetin (800, 1200 or 1600 μM). Control groups received 100 μL of SS-D or myricetin at a concentration of 1600 μM.

### 4.8. Intrinsic Fluorescence Experiments

The relative intrinsic fluorescence intensity of Batx-I with and without myricetin was monitored with a PerkinElmer spectrofluorometer (Waltham, MA, USA). The reaction mixture of 500 μL in a quartz cuvette (1 cm path length) contained 100 mM Tris HCl buffer (pH 7.4), Batx-I (1 μg/mL) and 150 μM of myricetin. Fluorescence spectra were measured between 300 and 500 nm after excitation at 280 nm. Three spectra were taken for each sample and all spectra were corrected by subtraction of buffer blanks.

### 4.9. Statistical Analysis

In order to determine significant differences between control and myricetin doses in the inhibition of proteolytic, hemorrhagic and myotoxic activities, an ANOVA followed by Tukey test was applied. Significant differences between control and myricetin in the inhibition of hemorrhagic activity with independent injection were determined by an unpaired Student’s *t*-test. For this study, p<0.05 was accepted as the level of significance. Results are shown as mean ± SEM (standard error of the mean), with the number of samples *n* indicated in each case.

### 4.10. Computational Studies

#### 4.10.1. Molecular Docking

The software Avogadro 1.90.0 [53] was used to build the myricetin structure and to optimize its conformation by an energy minimization process based on the MMF94 force field. Although the sequence of Batx-I is not completely characterized, portions of the sequence are identical to BaP1. Furthermore, the two SVMPs have comparable biological activities [46]. Therefore, the structure of the metalloproteinase BaP1 from *B. asper* (PDB code 2W15) [45] was used as a model toxin for the computational studies. The program AutoDock Vina (Scripps Research Institute, San Diego, CA, USA) [54] was used to dock myricetin into BaP1.

#### 4.10.2. Molecular Dynamics

The myricetin structure was parameterized with the CHARMM General Force Field using the ParamChem web interface [55,56]. The metalloproteinase was represented in the simulations by the CHARMM36m force field for proteins [57,58] and constructed using the server CHARMM-GUI [59,60]. Conventional molecular dynamics simulations were performed with NAMD [61] and analyzed with VMD (Visual Molecular Dynamics) [62]. Lennard–Jones interactions were calculated with a 12 Å cutoff, smoothly truncated beginning at 10 Å. The pressure was maintained at 1.01325 bar using the Langevin piston method. The temperature was maintained at 310.15 K using a Langevin thermostat with a damping parameter of 1 $ps^{-1}$. All simulations were performed with mass repartitioning of ligand and protein hydrogen atoms [63]. The mass distribution of water molecules was not altered. Electrostatic interactions were calculated using the particle-mesh Ewald method [64] with a grid spacing of 1.2 Å. Water molecules were represented by the TIP3P model [65]. Sodium and chloride ions (0.15 M NaCl) were added to the aqueous phase. Additional ions were added to obtain charge neutrality.

#### 4.10.3. Free Energy Calculations

The potentials of mean force were calculated by the adaptive biasing force method (ABF) [66,67] using the implementation provided in the Colvars module of NAMD [68]. In order to calculate the binding free energy of myricetin to the metalloproteinase BaP1 active site, the transition coordinate *Z* was defined as the distance along the *z*-axis of the center of mass of myricetin from the center of mass of the amino acids Asn106, Ile107, Thr139, His142, Glu143, His146, His152 and Ala167, located close to the binding site defined by docking calculations. The *z*-axis was established approximately through the center of mass of the amino acids Val123 to Leu144. After minimization and equilibration of the system, ABF was applied along the transition coordinate on the interval 2.0 ≤ *z* ≤ 20.0 Å. A harmonic restraint was applied when the distance between myricetin and the line defining the transition coordinate surpassed 5.0 Å, keeping the ligand within a cylinder of radius 5.0 Å. Force samples were collected in bins having widths of 0.05 Å. The standard binding free energy was calculated by:

(2) $$\Delta G^{{}^{\circ}}=-k_{B}T\ln\left(\pi R^{2}C_{0}\int dZ\;\exp\left[-\beta \;\Delta G\left(Z\right)\right]\right),$$

where $\beta =\frac{1}{k_{B}T}$ is the inverse thermal energy, R = 5 Å is the radius of the restraining cylinder and $C_{0}$ is the standard concentration (1/1660.5389Å${}^{3})$ [69]. Four independent calculations were performed using the simulation conditions previously described totaling 2.5 μs each of the systems.

## Supplementary Materials

The following are available online at http://www.mdpi.com/1420-3049/23/10/2662/s1, Figure S1: Purity of Batx-I isolated from *B. atrox* venom analyzed by SDS-PAGE under reduced conditions and reverse-phase high-performance liquid chromatography (RP-HPLC).

## Abbreviations

The following abbreviations are used in this manuscript:

ABF	Adaptive biasing force
Colvars	Collective variables
IC${}_{50}$	Concentration of myricetin where the enzyme activity is reduced by half
SS-D	1% dimethyl sulfoxide in sterile saline solution
SVMPs	Snake venom metalloproteinases
