# Supplementary material for: Inhibition of a Snake Venom Metalloproteinase by the Flavonoid Myricetin
Source: Molecules. 2018 Oct 16;23(10):2662. doi: 10.3390/molecules23102662 (PMC6222685; doi:10.3390/molecules23102662)
Supplement: Supplementary file 1 [file molecules-23-02662-s001.zip › molecules-359942-revise-supplementary.pdf]

Article

Inhibition of a Snake Venom Metalloproteinase by the Flavonoid Myricetin

Lina María Preciado [1*], Jeffrey Comer [2], Vitelbina Núñez [1,3], Paola Rey-Súarez [1], Jaime Andrés Pereañez [1*]

[1] Programa de Ofidismo/Escorpionismo, Facultad de Ciencias Farmacéuticas y Alimentarias, Universidad de Antioquia UdeA, Calle 70 No. 52-21, Medellín, Colombia.
[2] Institute of Computational Comparative Medicine, Kansas State University, Manhattan, Kansas, USA, 66506.
[3] Escuela de Microbiología, Universidad de Antioquia, UdeA, Calle 70 No. 52-21, Medellín, Colombia.
* Correspondence: maria.preciado@udea.edu.co; jaime.pereanez@udea.edu.co; Tel.: +57 (4) 219 6536

Academic Editor: name
Version September 21, 2018 submitted to Molecules; Typeset by LaTeX using class file mdpi.cls

---

Video S2: Minimum free-energy conformation of myricetin bound to BaP1.

© 2018 by the authors. Submitted to *Molecules* for possible open access publication under the terms and conditions of the Creative Commons Attribution license (http://creativecommons.org/licenses/by/4.0/)

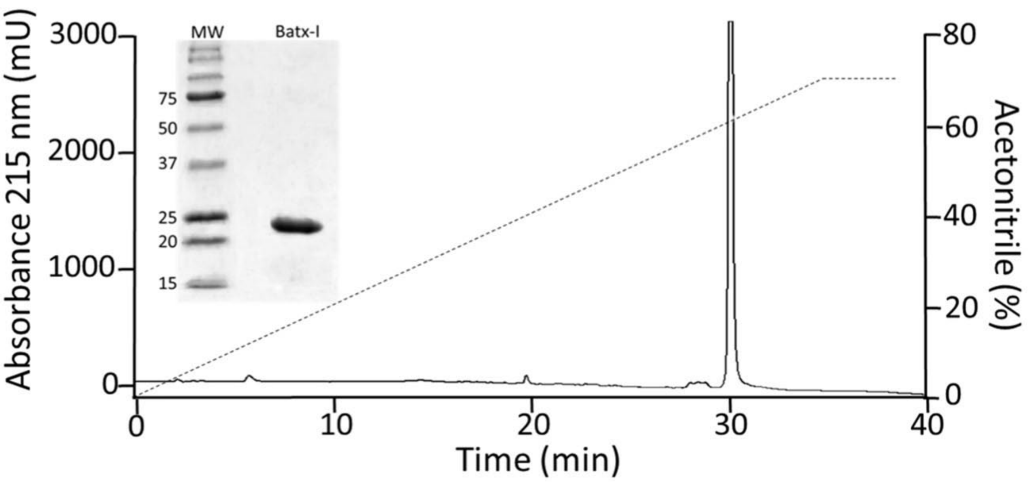

Figure S1. Purity of Batx-I isolated from *B. atrox* venom analyzed by SDS-PAGE under reduced conditions and reverse-phase high-performance liquid chromatography (RP-HPLC).
