# Supplementary figures and images for: Inhibition of a Snake Venom Metalloproteinase by the Flavonoid Myricetin
Source: Molecules. 2018 Oct 16;23(10):2662. doi: 10.3390/molecules23102662 (PMC6222685; doi:10.3390/molecules23102662)

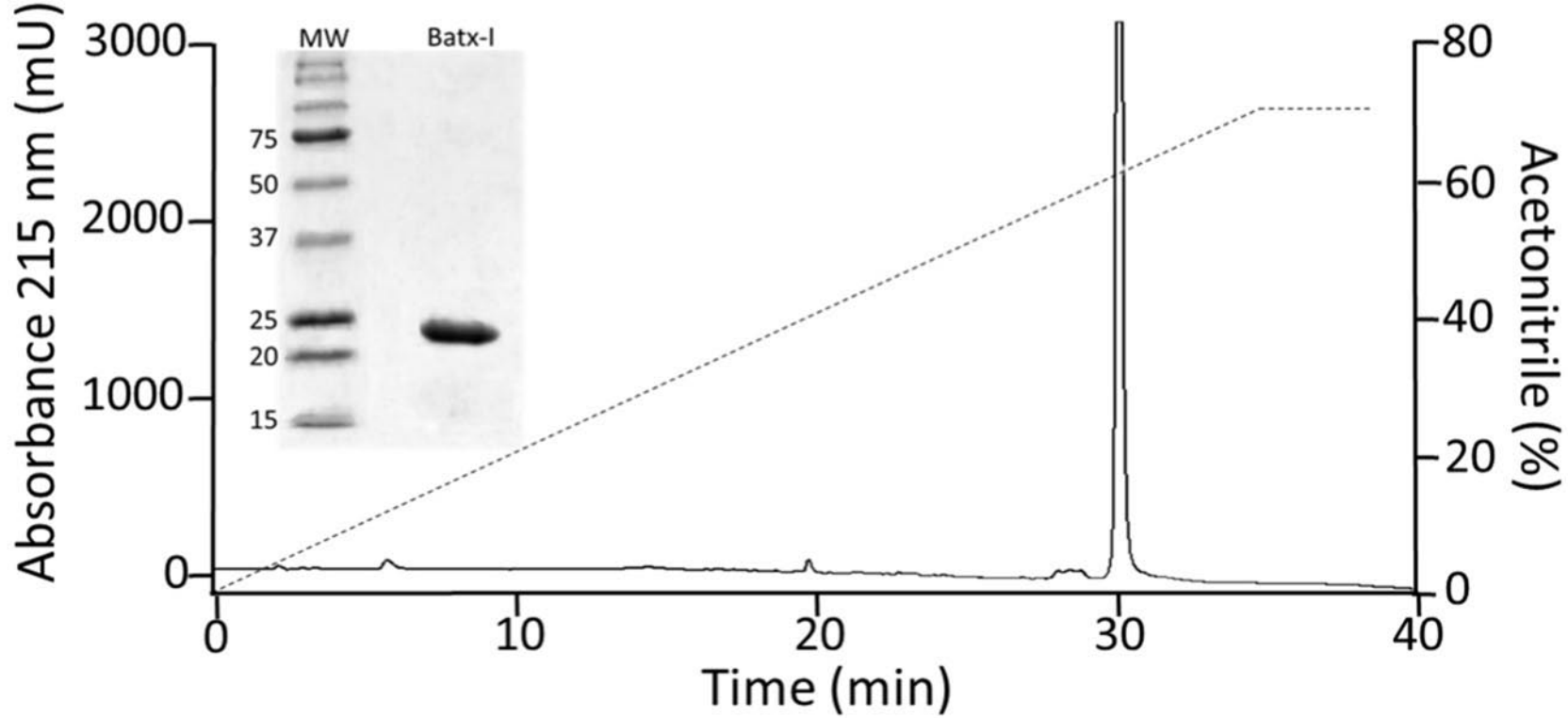

Supplement: Supplementary file 1 [file molecules-23-02662-s001.zip › Figure_S1.pdf]
